# Supplementary material for: Adapting COVID-19 research infrastructure to capture influenza and respiratory syncytial virus alongside SARS-CoV-2 in UK healthcare workers winter 2022/23: Results of a pilot study in the SIREN cohort
Source: PLoS One. 2025 May 22;20(5):e0316131. doi: 10.1371/journal.pone.0316131 (PMC12097552; doi:10.1371/journal.pone.0316131)
Supplement: Attachment — Submitted filename: Response to Reviewers.docx [file pone.0316131.s002.docx]

**Response to Reviewers**

Please see below responses to all comments provided by the academic editor and reviewer. Comments from the academic editor and reviewer are in **BOLD** and responses not in bold.

**As a potential benefit of using multiplex testing, if sample size allows please consider the value of analyzing or providing some information on patients with co-detection of viruses.**

Thank you for this comment, we have amended our analysis and updated the manuscript to now include data on PCR samples that were positive for more than one virus. However, for the symptom profile and trends by infection analysis, these samples have been excluded in order to compare across infections. A note has been added to the manuscript to explain this.

**Journal Requirements:**

**1. When submitting your revision, we need you to address these additional requirements.**

**Please ensure that your manuscript meets PLOS ONE's style requirements, including those for file naming.**

**We have reviewed and updated the manuscript to make sure it is written and formatted to the journal’s style requirements.**

**2. Thank you for stating the following** **financial disclosure: 
The SIREN study was funded by the UK Health Security Agency; the UK Department of Health and Social Care with contributions from the governments in Northern Ireland, Wales, and Scotland; the National Institute for Health Research; Health Data Research UK (NIHR200927; HDRUK2022.0322).
  
Please state what role the funders took in the study.  If the funders had no role, please state: "The funders had no role in study design, data collection and analysis, decision to publish, or preparation of the manuscript." 
If this statement is not correct you must amend it as needed. 
Please include this amended** **Role of Funder statement in your cover letter; we will change the online submission form on your behalf.**

**We have included in the below text in our updated covering letter, as a Role of Funder statement:**

**"****The funders had no role in study design, data collection and analysis, decision to publish, or preparation of the manuscript."**

**3. Thank you for stating the following in the** **Acknowledgments Section of your manuscript: 
Our thanks go to the participants in the SIREN study. We would also like to thank the help and support offered by the Berkshire Research Ethics Committee and the HDR-UK for winter pressures funding and to UKHSA for core SIREN study funding. Thanks also to all research team and site staff not mentioned above.**

**We note that you have provided funding information that is not currently declared in your Funding Statement. However, funding information should not appear in the Acknowledgments section or other areas of your manuscript. We will only publish funding information present in the Funding Statement section of the online submission form. 
Please remove any funding-related text from the manuscript and let us know how you would like to update your Funding Statement. Currently, your Funding Statement reads as follows: 
The SIREN study was funded by the UK Health Security Agency; the UK Department of Health and Social Care with contributions from the governments in Northern Ireland, Wales, and Scotland; the National Institute for Health Research; Health Data Research UK (NIHR200927; HDRUK2022.0322).**

**Please include your amended statements within your cover letter; we will change the online submission form on your behalf.**

**We have updated the acknowledgments section in the manuscript and in the updated covering letter.**

**4. In the online submission form, you indicated that Anonymised data will be made available for secondary analysis to trusted researchers upon reasonable request.**

**All PLOS journals now require all data underlying the findings described in their manuscript to be freely available to other researchers, either a. In a public repository, b. Within the manuscript itself, or c. Uploaded as supplementary information.
This policy applies to all data except where public deposition would breach compliance with the protocol approved by your research ethics board. If your data cannot be made publicly available for ethical or legal reasons (e.g., public availability would compromise patient privacy), please explain your reasons on resubmission and your exemption request will be escalated for approval.**

As per our ethics arrangements and in view of the importance of transparency with our study participants, we have agreed to maintain a centralised log of all researchers who have access to our data and samples. We communicate with our participants via newsletters and the study’s website of any new researchers who have access to the study data. In addition, we discuss any new analysis or potential research groups with our study’s participant involvement panel to access acceptability of the analysis/research group before going a head with any new projects. Before undertaking any new collaborations we are required to obtain a Data Sharing Agreement (DSA) as per our organisational legal requirement as data controller. Therefore, by providing a dataset along side this manuscript we would not be aware of the researcher(s) who would use the study’s data. We are happy to discuss further.

As per requested, this is the point of contact with regards to data restriction: [DataAccess@ukhsa.gov.uk](mailto:DataAccess@ukhsa.gov.uk)

**5. One of the noted authors is a group or consortium "SIREN Study Group". In addition to naming the author group, please list the individual authors and affiliations within this group in the acknowledgments section of your manuscript. Please also indicate clearly a lead author for this group along with a contact email address.**

**We have included the individual authors and affiliations of the SIREN study group into the acknowledgments section. We have updated in the manuscript and covering letter.**

**6. Please include your full ethics statement in the ‘Methods’ section of your manuscript file. In your statement, please include the full name of the IRB or ethics committee who approved or waived your study, as well as whether or not you obtained informed written or verbal consent. If consent was waived for your study, please include this information in your statement as well.**

**We have included an ethics statement into the method section of the manuscript.**

**Additional Editor Comments:

The Authors are expected to address all comments by the Reviewer. In particular, please provide the rationale of excluding samples with detection of multiple viruses, and describe the testing schedule in the Methods. In additional to the above comments, please address,**

**1. As a potential benefit of using multiplex testing, if sample size allows please consider the value of analyzing or providing some information on patients with co-detection of viruses.**

We have amended our analysis and updated the manuscript to now include data on PCR samples that were positive for more than one virus. However, for the symptom profile and trends by infection analysis, these samples have been excluded in order to compare across infections. A note has been added to the manuscript to explain this.

**Reviewers' comments:**

**Reviewer #1: I enjoyed reading the paper “Adapting COVID-19 research infrastructure to capture influenza and respiratory syncytial virus alongside SARS-CoV-2 in UK healthcare workers winter 2022/23: Results of a pilot study in the SIREN cohort”. Although the paper could use some more “focus” – I would suggest to use a couple of main topics and discuss them in every part of the paper in the same order. Consider to put more attention on RSV as that is new(er) information.

We have added additional detail on RSV in the introduction section, highlighted it in the results and discussion.**

**ABSTRACT

1.Please specify participants a bit further in abstract when possible “5,863 participants were included, 84.6% female, 70.3% ≥45-years, and 33.4% were nurses.”

We have added additional detail on demographics, occupation and vaccination into the abstract, to provide addition context and description about our cohort.**

**INTRODUCTION

2.The introduction does not read “easily” in my opinion as the authors do not introduce the three viruses & there epidemiology separately at the beginning. As far as I am aware RSV has a different seasonal variation and also a different vaccination approach. And the effect(s) of influenza (vaccination) in HCW (including sick leave & transmission) is probably much more investigated than described by the authors and more than for RSV & SARS-CoV-2.**

**We have included additional detail regarding the epidemiology, seasonal trends and vaccination on the three viruses. We have provided more focus on RSV as suggested by the reviewer.**

**3. Consider describing the status of RSV vaccination protocols in the UK**

**This has now been included in the introduction section in the manuscript.**

**METHODS

4.How many samples were positive for two or more viruses and why did the authors choose to exclude these? “samples which were positive for multiple viruses were excluded from the analysis”**

**We have updated our analysis and manuscript to include swabs that are positive for multiple viruses.**

**5.Describe the testing schedule (see my comment later at the discussion session)**

**We have stated the testing schedule in the methods section and added detail regarding the different number of test results for each virus into the discussion.**

**RESULTS

6.Main reasons for exclusion are missing**

**The exclusion criteria were included in Figure 1, however, we have now included in the results section, as suggested by the reviewer.**

**7.Please describe the study population in more detail especially the 70% who are not nurses in the results section**

**We have included additional detail on the demographic and occupation of the cohort into the results section.**

**8.Was there any effect of household composition? Especially having young children?

We have added a paragraph to the results section to describe the differences in household composition by infection.**

**DISCUSSION

9.Please mention this differences in testing schedules in the method section “A key limitation of this pilot study was the coverage and timing of multiplex PCR roll-out. There were fewer participants with PCR results for influenza and RSV than those with a SARS-Cov 2 test result”**

**Additional detail regarding the difference in the number of swabs tested for each virus has been added to the limitations section of the discussion.**

**10. I miss explanations or ideas concerning the study population which mostly consists of women and participants over 45 “Most participants were female (84.6%), of white ethnicity (91.4%), over 45-years of age (70.3%); nursing was the largest staff group (33.4%)”**

We have added a section in the discussion, comparing the SIREN cohort to the wider NHS workforce.
